# Identification of novel mutations and sequence variants in the *SOX2* and *CHX10* genes in patients with anophthalmia/microphthalmia

**Published:** 2008-03-24

**Authors:** Jie Zhou, Femida Kherani, Tanya M. Bardakjian, James Katowitz, Nkecha Hughes, Lisa A. Schimmenti, Adele Schneider, Terri L. Young

**Affiliations:** 1 Division of Ophthalmology, Children’s Hospital of Philadelphia, Philadelphia, PA; 2 Division of Genetics, Children’s Hospital of Philadelphia, Philadelphia, PA; 3 Division of Genetics, Albert Einstein Medical Center, Philadelphia, PA; 4 Department of Pediatrics and Ophthalmology, Institute of Human Genetics, University of Minnesota Minneapolis, MN

## Abstract

**Purpose:**

Mutations in the *SOX2* and *CHX10* genes have been reported in patients with anophthalmia and/or microphthalmia. In this study, we evaluated 34 anophthalmic/microphthalmic patient DNA samples (two sets of siblings included) for mutations and sequence variants in *SOX2* and *CHX10*.

**Methods:**

Conformational sensitive gel electrophoresis (CSGE) was used for the initial *SOX2* and *CHX10* screening of 34 affected individuals (two sets of siblings), five unaffected family members, and 80 healthy controls. Patient samples containing heteroduplexes were selected for sequence analysis. Base pair changes in *SOX2* and *CHX10* were confirmed by sequencing bidirectionally in patient samples.

**Results:**

Two novel heterozygous mutations and two sequence variants (one known) in *SOX2* were identified in this cohort. Mutation c.310 G>T (p. Glu104X), found in one patient, was in the region encoding the high mobility group (HMG) DNA-binding domain and resulted in a change from glutamic acid to a stop codon. The second mutation, noted in two affected siblings, was a single nucleotide deletion c.549delC (p. Pro184ArgfsX19) in the region encoding the activation domain, resulting in a frameshift and premature termination of the coding sequence. The shortened protein products may result in the loss of function. In addition, a novel nucleotide substitution c.*557G>A was identified in the 3′-untranslated region in one patient. The relationship between the nucleotide change and the protein function is indeterminate. A known single nucleotide polymorphism (c. *469 C>A, SNP rs11915160) was also detected in 2 of the 34 patients. Screening of *CHX10* identified two synonymous sequence variants, c.471 C>T (p.Ser157Ser, rs35435463) and c.579 G>A (p. Gln193Gln, novel SNP), and one non-synonymous sequence variant, c.871 G>A (p. Asp291Asn, novel SNP). The non-synonymous polymorphism was also present in healthy controls, suggesting non-causality.

**Conclusions:**

These results support the role of *SOX2* in ocular development. Loss of *SOX2* function results in severe eye malformation. *CHX10* was not implicated with microphthalmia/anophthalmia in our patient cohort.

## Introduction

Anophthalmia (an absence of eye structures) and/or microphthalmia (an abnormally small eye) are rare disorders with a prevalence of 0.2–0.4 per 10,000 births in developed countries [1-3]. One or both eyes are affected in isolation or as part of other birth defects [4-9]. The disease exhibits diverse patterns of genetic inheritance, and the severity is variable due to the genetic heterogeneity of the ocular malformation.

Mutations in several human genes are associated with anophthalmia and microphthalmia. Among them, mutations in *SOX2* appear to account for most cases [4,10-14]. *SOX2*, which is located at chromosome 3q26.3-q27, encodes 317 amino acids that belong to the high-mobility-group (HMG) DNA-binding protein family. Expressed in embryonic stem cells and a wide variety of tissues during early development, SOX2 plays an important role in cell differentiation and early organogenesis [15,16]. In ocular tissues, the inhibition of *SOX2* expression in the developing *Xenopus* retina has been shown to reduce cell proliferation and allows cells to develop into non-neuronal cell types [17]. Working in concert with PAX6, SOX2 regulates downstream target genes such as the crystalline genes to guide early lens development [18].

Mutations in *SOX2* account for approximately 10% of anophthalmia and microphthalmia cases [4,10,19]. Previously reported cases of anophthalmia and microphthalmia were associated with *SOX2* whole-gene deletions or coding sequence mutations [4,10-13,19,20]. In this study, a cohort of 34 anophthalmia/microphthalmia patients was screened for mutations in *SOX2*. Two novel heterozygous mutations and two sequence variants (one novel, one known SNP) were identified in 6 (two siblings) out of 34 patients. This confirms the importance of *SOX2* mutation screening in patients with anophthalmia/microphthalmia. In screening unaffected family members, we also found an individual with a pathogenic allele suggesting the novel finding that autosomal dominant *SOX2* mutations can be non-penetrant.

As part of our large scale screening campaign, mutation analysis of *CHX10* was also performed. The homeodomain protein CHX10 has been shown to be essential for ocular development [21], and autosomal recessive mutations of the gene have been implicated in microphthalmia in both humans and mice [22-24]. Screening of the same cohort of anophthalmia/microphthalmia patients identified two synonymous sequence variants in exon 3 and one non-synonymous sequence variant in exon 5. This non-synonymous polymorphism was also present in normal controls. We did not find sequence variants in other exons of *CHX10*, suggesting that *CHX10* mutations are less frequent in patients with anophthalmia.

## Methods

### Subjects

To identify existing and potentially novel mutations of *SOX2* and *CHX10* in a cohort of anophthalmia and microphthalmia patients, we undertook a candidate gene screening approach. We screened 34 affected individuals (two sets of siblings), five unaffected family members, and 80 healthy controls. Patient demographics are summarized in Appendix 1. The study was approved by the Institutional Review Board for Human Subject Research at the Children’s Hospital of Philadelphia and at the Albert Einstein Medical Center. The study also conformed to the tenets of the Declaration of Helsinki. Informed consent was obtained from all subjects whose blood and DNA samples were used in the analysis. Peripheral blood samples (10–20 ml) were collected, and genomic DNA was extracted using the Puregene DNA isolation kit (Gentra Systems, Minneapolis, MN).

### Conformation-sensitive-gel-electrophoresis

Conformation-sensitive-gel-electrophoresis (CSGE) was used for initial *SOX2* and *CHX10* gene screenings. Standard polymerase chain reaction (PCR) was performed on genomic DNA of affected patients, unaffected relatives, and healthy controls to amplify *SOX2* and *CHX10* exonic sequences (primer sets are available upon request). An additional 120 base pairs of intronic sequences at both the 5′-end and 3′-end were also included. PCR amplification was performed in a reaction volume of 25 μl containing 50 ng of genomic DNA, 200 μM dNTP, 0.4 μM of each primer pair, 1.5 mM MgCl_2_, 1X PCR buffer, 1X Q-solution, and 1.25 units of *Taq* polymerase (Qiagen, Valencia, CA). A single annealing temperature of 55 °C was used for all primers. PCR products were separated by electrophoresis in an 8% polyacrylamide gel for detection of heteroduplexes and homoduplexes. Patient samples containing heteroduplexes were selected for sequence analysis. Base pair changes in *SOX2* and *CHX10* were confirmed by sequencing bidirectionally in patient samples.

## Results

**Figure 1:**
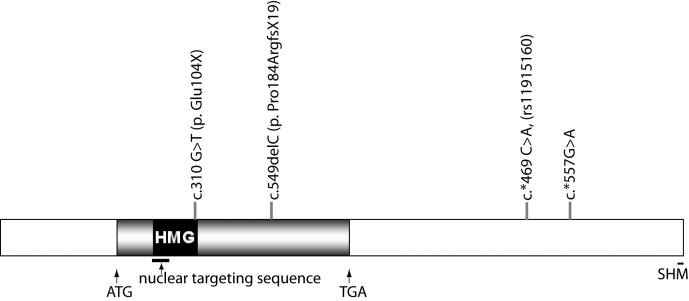
*SOX2* gene structure and mutations in anophthalmia/microphthalmia. The 5′- and 3′- untranslated regions are shown as blank boxes, and the coding region is shown as a filled box. The start (ATG) and the stop (TGA) codons are indicated. Mutations detected in this study are indicated. The nuclear targeting sequence is underlined. HMG=high mobility group, G=guanosine, T=thymidine, C=cytidine, A=adenosine, Glu=glutamic acid, Pro=proline, Arg=arginine, X=stop, FS=frameshift.

Of the 34 patients (32 independent probands) analyzed, three (two siblings) showed novel mutations in coding sequence and three had two sequence variants in the 3′-untranslated region (UTR) of *SOX2* (Figure 1 and Table 1). Mutation, c.310 G>T (p. Glu104X), found in one patient, was in the region encoding the HMG DNA-binding domain and resulted in a change from glutamic acid to a stop codon. The second mutation, noted in two affected siblings, was a single nucleotide deletion c.549delC (p. Pro184ArgfsX19) in the region encoding the activation domain, resulting in a frameshift and premature termination of the coding sequence. In addition, a novel nucleotide substitution, c.*557G>A, was identified in the 3′-untranslated region in one patient, and a known single nucleotide polymorphism (c. *469 C>A, SNP rs11915160) was also detected in 2 out of 34 patients (Appendix 1).

**Table 1:** Sequence variations in *SOX2*.

**ID**	**Fragment 4**	**Sequence results**	**Fragment 5**	**Sequence results**	**Fragment 8**	**Sequence results**	**Fragment 9**	**Sequence results**
012A	homoduplex		homoduplex		heteroduplex	*469C>A^1^	homoduplex	
013A	homoduplex		homoduplex		heteroduplex	*469C>A^1^	homoduplex	
018A	homoduplex		homoduplex		homoduplex		homoduplex	
023A	homoduplex		homoduplex		homoduplex		homoduplex	
023I	homoduplex		homoduplex		homoduplex		homoduplex	
025A	homoduplex		homoduplex		homoduplex		homoduplex	
027A	homoduplex		homoduplex		homoduplex		homoduplex	
028A	homoduplex		homoduplex		homoduplex		homoduplex	
029A	homoduplex		homoduplex		homoduplex		homoduplex	
030A	homoduplex		homoduplex		homoduplex		homoduplex	
031A	homoduplex		homoduplex		homoduplex		homoduplex	
033A	homoduplex		homoduplex		homoduplex		homoduplex	
034A	homoduplex		homoduplex		homoduplex		homoduplex	
035A	homoduplex		homoduplex		homoduplex		homoduplex	
037A	homoduplex		homoduplex		homoduplex		homoduplex	
038A	homoduplex		homoduplex		homoduplex		homoduplex	
040A	heteroduplex	310G>T	homoduplex		homoduplex		homoduplex	
041A	homoduplex		homoduplex		homoduplex		homoduplex	
042A	homoduplex		homoduplex		homoduplex		homoduplex	
043A	homoduplex		homoduplex		homoduplex		homoduplex	
044A	homoduplex		homoduplex		homoduplex		homoduplex	
045A	homoduplex		homoduplex		homoduplex		homoduplex	
046A	homoduplex		homoduplex		homoduplex		homoduplex	
047A	homoduplex		homoduplex		homoduplex		homoduplex	
048A	homoduplex		homoduplex		homoduplex		homoduplex	
049A	homoduplex		homoduplex		homoduplex		heteroduplex	*557G>A
050A	homoduplex		homoduplex		homoduplex		homoduplex	
051A	homoduplex		homoduplex		homoduplex		homoduplex	
052A	homoduplex		homoduplex		homoduplex		homoduplex	
053D	homoduplex		homoduplex		homoduplex		homoduplex	
053A	homoduplex		homoduplex		homoduplex		homoduplex	
054A	homoduplex		quadruplet	549delC	homoduplex		homoduplex	
054H	homoduplex		triplet	549delC	homoduplex		homoduplex	
055A	homoduplex		homoduplex		homoduplex		homoduplex	

The entire genomic sequence of *SOX2* was screened by CSGE. Samples which show heteroduplex, triplet, and quadruplet on CSGE are sequenced bi-directionally. Homoduplex indicates two identical alleles. Heteroduplex, triplet, and quadruplet indicate different alleles. One allele is the wild type sequence and the other one is a sequence variant differing from the wild type one. Novel mutation at c.310 G>T results in a nonsense amino acid change at p. Glu104X. The novel single nucleotide deletion at c.549delC results in a frame shift mutation and premature termination at 19 amino acids downstream of the deletion site (p.Pro184ArgfsX19). The novel single nucleotide substitution at c.*557G>A is in the 3′-UTR and does not predictably change the SOX2 protein. Another single nucleotide substitution at *469 C>A is a known SNP (rs11915160) located in the 3′-UTR. G=guanosine, T=thymidine, C=cytidine, A=adenosine, Glu=glutamic acid, Pro=proline, Arg=arginine, X=Stop, FS=frameshift; ^1^SNP rs11915160

Patient 40A is an eight-year-old child with bilateral anophthalmia. Clinical features included dilated ventricles with ventriculo-peritoneal shunt placement for hydrocephalus, microcephaly, atrophic optic nerves, chiasm, posterior corpus callosum and splenium, and abnormal electroencephalograms with no overt seizures. The patient has short stature and delayed motor and mental development. He has an unusual ataxic gait but is independently mobile with a walker. He has begun to speak in three to four word sentences.

**Figure 2:**
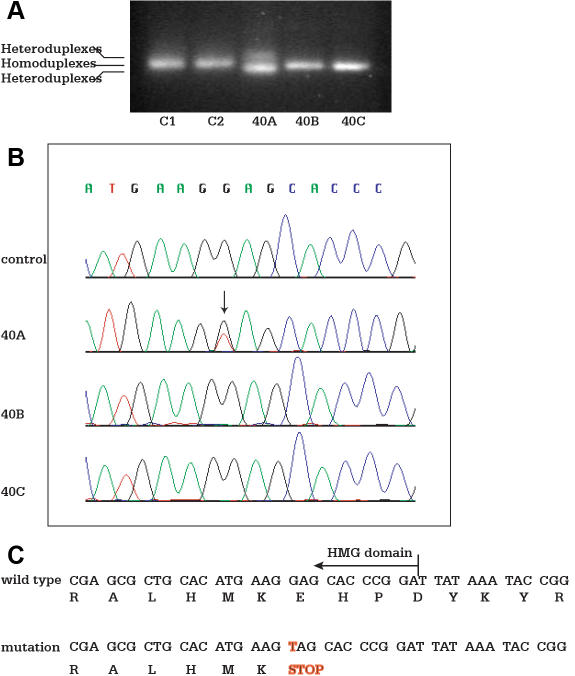
Mutation in the *SOX2* HMG domain. Genomic DNA from affected patients, unaffected relatives, and healthy controls are amplified by PCR. PCR products are separated by electrophoresis in an 8% polyacrylamide gel for detection of heteroduplexes and homoduplexes. Genomic DNA samples are then sequenced for detection of sequence variants. **A**: Heteroduplexes detected by CSGE is shown. Lanes C1 and C2 represent the healthy controls; Lane 40A represents the proband; Lane 40B represents the mother of the proband; and Lane 40C is the father of the proband. Sample from patient 40A shows heteroduplex comparing to homoduplex observed in samples from the parents (40B and 40C) and normal controls (C1 and C2). **B:** Single nucleotide substitution at c.310 G>T of the coding sequence is identified in patient 40A, but not in the parents (40B and 40C) and normal controls (C1 and C2). **C:** Mutation at c.310 G>T results in a nonsense amino acid change at p. Glu104X as illustrated. HMG=high mobility group.

The amplified genomic fragments from the patient (40A) showed faster and slower migrating products comparing to that of the wild type observed in samples from the parents (40B and 40C) and normal controls (C1 and C2) with CSGE (Figure 2A). The heteroduplexes suggest a mutation in the fragment. The genomic DNA from this patient was sequenced bidirectionally. Two alleles were identified (Figure 2B), one with a wild type allele and one with a nucleotide substitution at c.310 G>T of the coding sequence, resulting in a nonsense amino acid change (p. Glu104X). The predicted shorter peptide is truncated at the end of the HMG domain, which results in the deletion of the entire COOH-terminal activation domain (Figure 2C). The mutation, located in the HMG domain, is not directly involved in DNA binding based on NMR-spectroscopy structure from the Protein Data Bank (PDB). Both parents were homozygous for the wild type allele.

**Figure 3:**
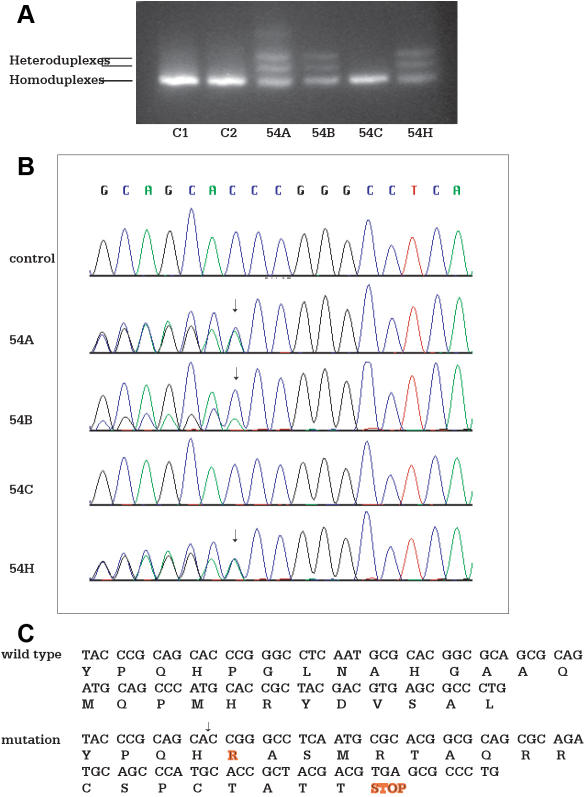
Mutation in the *SOX2* activation domain. Genomic DNA from affected patients, unaffected relatives, and healthy controls are amplified by PCR. PCR products are separated by electrophoresis in an 8% polyacrylamide gel for detection of heteroduplexes and homoduplexes. Genomic DNA samples are then sequenced for detection of sequence variants. **A**: Heteroduplexes detected by CSGE is shown. Lanes C1 and C2 represent the healthy controls; Lane 54A represents the proband; Lane 54B represents the mother of the proband; Lane 54C represents the father of the proband; and Lane 54H represents the affected sibling of the proband. The additional slower band in patient 54A may represent a conformational intermediate formed during gel electrophoresis, resulting in a different mobility compared to homoduplexes and the triplet. **B:** Sequence analysis shows two alleles in the proband 54A, the affected sibling 54H, and the clinically normal mother 54B. One allele is the wild type sequence and the other one is single nucleotide deletion at c.549delC. The father 54C is homozygous for the wild type allele. **C:** The single nucleotide deletion at c.549delC results in a frame shift mutation and premature termination at 19 amino acids downstream of the deletion site (p.Pro184ArgfsX19). The mutation is predicted to delete part of the C-terminal activation domain and to produce a truncated peptide.

Patient 54A is a nine-year-old child with right anophthalmia and left microphthalmia. Her clinical features included a right middle fossa arachnoid cyst and partial absence of the posterior aspect of the corpus callosum including the splenium. She had mild hydrocephalus and partial complex seizures. Other abnormalities were growth and thyroid hormone deficiency, developmental delays, and a wide-based ataxic gait. She also presented with facial asymmetry with a beaked nose, small widely-spaced teeth, and diastasis recti. The parents were unaffected. CSGE analysis showed three slower migrating product bands in addition to the wild type, indicative of heterozygosity (Figure 3A). The affected sibling of the proband (54H), who was diagnosed with bilateral anophthalmia by ultrasound in utero, had two slower migrating bands and the wild type product. An amniocentesis was performed, and a chromosome study was that of a normal female with a 46XX karyotype. Her birthweight of 8 lbs 15 ounces and length of 52 cm are appropriate for a term infant. Her newborn hearing screening test was normal. An orbital ultrasound shortly after birth revealed the absence of globe structures bilaterally. A head MRI at age of four months showed partial agenesis of the corpus callosum and hypoplasia of the orbits. Minimal ocular tissue was noted in the orbit with visualization of the extraocular muscles and lacrimal glands. The optic nerves were not visualized. The maxillae were hypoplastic.

At approximately nine months, she began treatment with thyroid supplementation for hypothyroidism. She is of a low growth percentile with normal growth hormone levels. She had urticaria pigmentosa at 14 months of age. At the age of 16 months, she sat without support, rolled, and babbled with consonants and vowels. She finger feeds and has no feeding difficulties. Her development is delayed but is more advanced than that of her sister, the proband, at the equivalent age. The mother has normal vision and an unremarkable ophthalmologic examination. She has normal motor abilities and intellect. A head MRI scan of the mother was unremarkable.

Sequence analysis detected two alleles in the proband, the affected sibling, and the clinically normal mother (54B). One allele was the wild type sequence and the other was a c.549delC (p.Pro184ArgfsX19) in the coding sequence (Figure 3B). The deletion changes the reading frame and results in a premature stop codon 19 amino acids downstream of the deletion site. The mutation is predicted to delete part of the COOH-terminal activation domain and to produce a truncated peptide (Figure 3C). The father (54C) of the proband was homozygous for the wild type allele.

**Figure 4:**
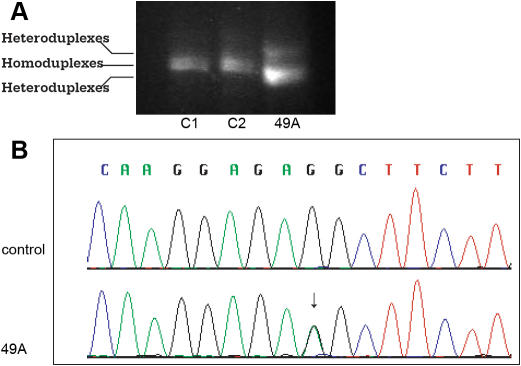
*SOX2* mutation in the 3′- untranslated region (3′-UTR). Genomic DNA from the affected patient and healthy controls are amplified by PCR. PCR products are separated by electrophoresis in an 8% polyacrylamide gel for detection of heteroduplexes and homoduplexes. Genomic DNA samples are then sequenced for detection of sequence variants. **A**: Heteroduplexes detected by CSGE is shown. Lanes C1 and C2 represent the healthy controls and Lane 49A represents the proband. Sample from patient 49A shows heteroduplex comparing to homoduplex observed in samples from normal controls (C1 and C2). **B:** A nucleotide substitution at c.*557G>A of the 3′-UTR is identified in patient 49A, but not in normal controls (C). The sequence variant does not predictably change the SOX2 protein and is not a previously described SNP.

Patient 49A is a 17-year-old female with bilateral anophthalmia. Her clinical features include a ventricular-septal defect, which resolved without surgical intervention, and fusion of two primary teeth of the left lower jaw. She is developmentally normal and is currently applying to four-year colleges. The parents were unaffected. A heterozygous sequence variant was identified in this individual in the 3′-UTR by CSGE (Figure 4A). Sequence analysis detected two alleles, one with a wild type sequence and one with a nucleotide substitution, c.*557G>A (Figure 4B). The sequence alteration does not predictably change the SOX2 protein and is not a previously described SNP (dbSNP Build 128). A review of transcription factor databases did not uncover known regulatory elements associated with the nucleotide substitution. DNA was unavailable from additional unaffected family members of the proband.

To determine that the above sequence alterations detected in anophthalmia and microphthalmia patients were not single nucleotide polymorphisms (SNPs), 80 healthy control individuals were examined by direct sequencing analysis. None of the above sequence alterations were found in the normal control subjects. During this screening, we also detected SNP c.*469 C>A in 2 out of 34 patients. The SNP is a known variant and located at 3′ UTR (SNP rs11915160).

To evaluate sequence variations in *CHX10*, the same cohort of patients were screened by CSGE and direct sequencing. The majority of sequence variations were detected in exon 3 (Table 2), and the mutations were all synonymous, c.471 C>T (p.Ser157Ser, rs35435463) and c.579 G>A (p. Gln193Gln, novel SNP). Another sequence variation was found in exon 5, c.871 G>A (p. Asp291Asn, novel SNP), in one affected individual resulting in a non-synonymous polymorphism. Using healthy controls, the same sequence variant was found, suggesting non-causality.

**Table 2:** Sequence variations in *CHX10*.

**Person ID**	**Exon 3**	**Sequence results**	**Exon 5**	**Sequence results**
012A	homoduplex		homoduplex	
013A	homoduplex		homoduplex	
018A	heteroduplex	c.471 C>T (p.S157S)	homoduplex	
023A	homoduplex		homoduplex	
023I	homoduplex		homoduplex	
025A	heteroduplex	c.471 C>T (p.S157S)	homoduplex	
027A	heteroduplex	c.471 C>T (p.S157S)	homoduplex	
029A	heteroduplex	c.471 C>T (p.S157S)	homoduplex	
030A	heteroduplex	c.471 C>T (p.S157S)	homoduplex	
031A	heteroduplex	c.471 C>T (p.S157S)	homoduplex	
033A	homoduplex		homoduplex	
034A	homoduplex		homoduplex	
035A	heteroduplex	c.471 C>T (p.S157S)	homoduplex	
037A	homoduplex		homoduplex	
038A	homoduplex		homoduplex	
040A	homoduplex		homoduplex	
041A	homoduplex		homoduplex	
042A	heteroduplex	c.471 C>T (p.S157S)	homoduplex	
043A	heteroduplex	c.471 C>T (p.S157S)	homoduplex	
044A	homoduplex		homoduplex	
045A	homoduplex		homoduplex	
046A	homoduplex		homoduplex	
047A	homoduplex		homoduplex	
048A	homoduplex		homoduplex	
049A	heteroduplex	c.579 G>A (Q193Q)	homoduplex	
050A	homoduplex		homoduplex	
051A	homoduplex		homoduplex	
052A	homoduplex		homoduplex	
053A	heteroduplex	c.471 C>T (p.S157S)	homoduplex	
054A	homoduplex		homoduplex	
054H	heteroduplex	c.471 C>T (p.S157S)	homoduplex	
055A	homoduplex		heteroduplex	c.871 G>A (p.D291N)*
056A	homoduplex		homoduplex	
058A	homoduplex		homoduplex	
059A	heteroduplex	c.471 C>T (p.S157S)	homoduplex	
061A	homoduplex		homoduplex	

All sequence variations detected in *CHX10* were in exons 3 and 5. Homoduplex indicates two identical alleles. Heteroduplex indicates different alleles. One allele is the wild type sequence and the other one is a sequence variant differing from the wild type one. The single nucleotide substitution at c.471 C>T is a known SNP (rs35435463) and results in a synonymous change in the protein sequence (p.Ser157Ser). The single nucleotide substitution at c.579 G>A is a novel SNP and results in a synonymous change in the protein sequence (p. Gln193Gln). Another novel sequence variation, c.871 G>A, is identified in exon 5 in one affected individual and results in a non-synonymous change in the protein sequence (p. Asp291Asn). This sequence variation is also identified in healthy controls. G=guanosine, T=thymidine, C=cytidine, A=adenosine, S=Ser=serine, Q=Gln=glutamine, D=Asp=aspartic Acid, N=Asn=asparagine. The asterisk indicates that the heteroduplex is detected in 4 of 50 healthy control samples.

## Discussion

The present study identified two novel mutations and two sequence variants (one novel, one known SNP) in *SOX2* in 6 (two siblings) out of 34 anophthalmia/microphthalmia/ coloboma patients. The frequency of mutations in *SOX2* in anophthalmia and microphthalmia patients is estimated at 10% for this study, which is consistent with previous reports [4,14,19]. A mutational analysis of *CHX10* of the same cohort of anophthalmia/microphthalmia patients identified two synonymous sequence variants in exon 3 and one non-synonymous sequence variant in exon 5. The non-synonymous polymorphism was also present in normal controls, suggesting it is not causative. Further sequence analysis of other genes associated with anophthalmia and microphthalmia such as *PAX6* [7,25], *BCOR* [26], *OTX2* [27], *SIX6* [28,29], and *CHD7* [30,31] is ongoing in this study cohort to determine mutation associations.

Two mutations, a nonsense mutation and a frameshift mutation, were located in the coding region of *SOX2*, and both resulted in truncation of the SOX2 protein. The shortened protein product may result in loss of function. In animal studies, homozygous mice for *SOX2* null allele are embryonic lethal [16] and heterozygous mice with one null allele are normal compared to that of the wild type [32]. Further reduction of the level of *SOX2* expression by deleting a neural cell-specific enhancer on a heterozygous background resulted in reduced viability and neurodegeneration [32]. These observations suggest that the abnormal phenotype may become apparent when *SOX2* expression falls below a certain threshold, or loss of function in one allele reduces *SOX2* expression below that level. One of the mutations detected in this study was at the 3′ UTR. Although the relationship between the mutation at the 3′-UTR and anophthalmia/ microphthalmia is indeterminate, one possibility is that the nucleotide substitution at c.*557G>A affects *SOX2* expression. This is supported by the observation that the 3′UTR of *SOX2* contains regulatory elements that enhance its transcriptional activity in embryonic stem cells [33].

Of interest is the transmission of a *SOX2* mutation from an unaffected mother to her two daughters in the example of sibling patients 54A and 54H. Gonosomal mosaicism, evidenced by the presence of a mutation in buccal cells but not in white cell-derived DNA, has been reported for *SOX2* in an unaffected parent [34]. We were able to identify this mutation in genomic DNA derived from white blood cells in the unaffected mother. This suggests that this mother may not be mosaic in this circumstance. We propose that the anophthalmia phenotype appears to be non-penetrant. Non-penetrant mutations have been reported previously in sonic hedgehog (SHH) [34] and in fibroblast growth factor receptor 1 (FGFR) [35]. This example underscores the importance of testing seemingly unaffected parents once a mutation is discovered in their offspring regardless of their phenotype and points out that some individuals with a *SOX2* mutation may be non-penetrant.

Our patients with coding region mutations had *SOX2* phenotypes consistent with previous studies [4,10-12]. All patients with mutations had bilateral anophthalmia and unilateral severe microphthalmia (40A, 49A, 54H) with the exception of patient 54A who had unilateral anophthalmia instead of bilateral anophthalmia. This suggests that *SOX2* mutations cause a more severe disruption of normal eye development and that microphthalmia/anophthalmia are of a spectrum of the same disease. The patients with coding sequence mutations (40A, 54A, and 54H) had significant brain abnormalities. Patient 40A showed dilated ventricles and microcephaly. Patient 54A was diagnosed with a right middle fossa arachnoid cyst and partial absence of posterior aspect of corpus callosum including the splenium. Patient 54H, the sibling of patient 54A, had partial agenesis of the corpus callosum. Brain structure changes are also consistent with other studies and are supported by murine models of *SOX2* deficiency with neurodegeneration and impaired brain neurogenesis [32]. Hormonal deficiencies and growth retardation are also important phenotypic associations with *SOX2* coding sequence changes (patients 40A, 54A, and 54H). Ragge et al. [10] have reported a case of anophthalmia/microphthalmia caused by a frameshift mutation in *SOX2* (case 9, c.628delA, p.Met210fs211X). The genetic location of that mutation is similar to the proband 54A and her sibling 54H (c.549delC, p.Pro184ArgfsX19) in this study. The neurological phenotypes in this study, including brain malformations and seizure activity, were also present in their case [10]. Patient 49A with a 3′-UTR mutation had a cardiac ventricular-septal defect with dental anomalies. Different forms of craniofacial and dental defects were also noted in some patients with coding region mutations of *SOX2* gene. Patient 54A (Appendix 1) had facial asymmetry and small, widely spaced teeth. One of the patients (case #1) reported by Ragge et al. [10] also showed craniofacial dysmorphisms and widely spaced teeth. The *SOX2* UTR mutations appear to be associated with severe eye development but are perhaps less likely to manifest with neurological manifestations.

In conclusion, this study confirms that heterozygous loss of function mutations in *SOX2* cause anophthalmia and microphthalmia and represent approximately 10% of patients ascertained for anophthalmia and microphthalmia but may also be non-penetrant. Mutations in *CHX10* are less frequently found. Genetic screening of other candidate genes and the identification of genetic factors influencing *SOX2* expression may help to determine the susceptibility of various disease alleles in the occurrence of this severe form of eye malformation and also help to identify factors that may influence *SOX2* expression. Further characterization of genetic interactions among causative genes may also help to further our understanding of the formation of the eye.

## Appendix 1. Patient demographics and mutations and sequence variants in *SOX2* and *CHX10* genes.

To access the data, click or select the words “Appendix 1”. This will initiate the download of a compressed (.zip) archive that contains the file. This file should be uncompressed with an appropriate program (the particular program will depend on your operating system).
